# Essential Oil Composition and Stable Isotope Profile of *Osmorhiza occidentalis* Torr. (Apiaceae) from Utah

**DOI:** 10.3390/plants11202685

**Published:** 2022-10-12

**Authors:** Tyler M. Wilson, Brett J. Murphy, Emma A. Ziebarth, Ariel Poulson, Chris Packer, Richard E. Carlson

**Affiliations:** D. Gary Young Research Institute, Lehi, UT 84043, USA

**Keywords:** Apiaceae, essential oil, gas chromatography, *Osmorhiza occidentalis*, stable isotope, yield

## Abstract

*Osmorhiza occidentalis* Torr. is an essential-oil-bearing plant in the Apiaceae family. Volatile oil was produced through steam distillation (*n* = 3) of the above ground plant parts and was analyzed by gas chromatography (GC/FID, GC/MS), and gas chromatography/isotope ratio mass spectrometry (GC/IRMS) to establish the essential oil composition and stable isotope profile. The resulting essential oils were found to be comprised of 33 volatile compounds. Prominent volatile compounds include methyl chavicol (avg. 61.6%), (*Z*)-β-ocimene (avg. 14.7%), sabinene (avg. 10.5%), and γ-terpinene (avg. 2.8%). Stable isotope values were determined for prominent volatile compounds, including methyl chavicol, (*Z*)-β-ocimene, sabinene, and γ-terpinene. Values for *δ*^2^H range from −393.479 (avg. sabinene) to −171.516 (avg. methyl chavicol). Those for *δ*^13^C range from −35.957 (avg. methyl chavicol) to −30.820 (avg. (*Z*)-β-ocimene). The essential oil yield was 0.12% (*w/w*). The current study establishes for the first time, to the best knowledge of the authors, the essential oil yield, essential oil composition, and stable isotope profile of prominent volatile compounds extracted from the above-ground portions of *O. occidentalis*. These results provide insight into the volatile chemical composition produced by the plant and provide fundamental data for substantiation of ethnobotanical applications.

## 1. Introduction

**Figure 1:**
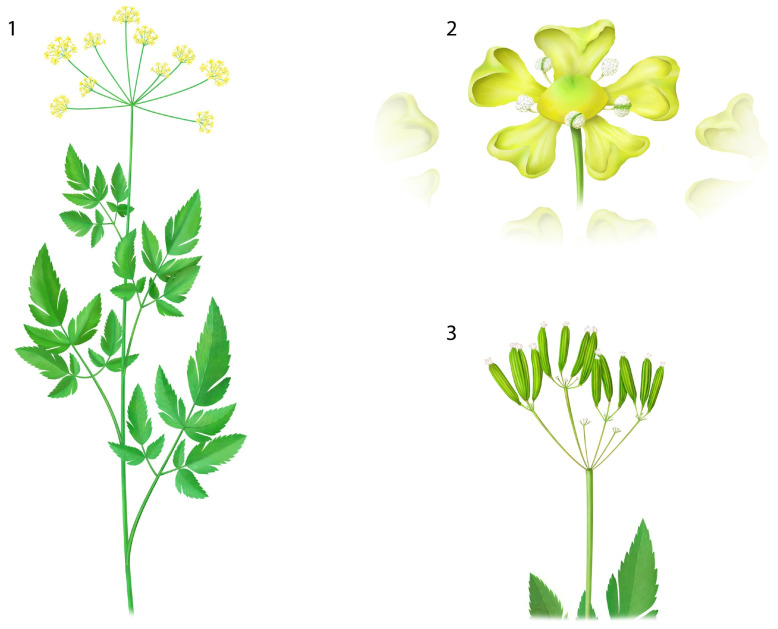
Botanical illustration of *Osmorhiza occidentalis* Torr. showing (1) above ground portions of the flowering plant, (2) close perspective of individual floret, and (3) plant bearing fruit. Illustrated by Rick Simonson, Science Lab Studios, Inc. (Kearney, NE, USA).

*Osmorhiza occidentalis* Torr. is an aromatic plant in the Apiaceae family (Figure 1) [1]. The genera name has Greek roots meaning ‘odorous root’ (*osme* = odor; *rhiza* = root) [2,3] and this species is commonly described as having roots and foliage with a licorice-like aroma [2,3,4]. *O. occidentalis* is native to the Intermountain Region, west into the Pacific Northwest (USA), and north into Canada [2,3,4,5,6,7,8,9,10].

Previous research has been conducted on seed germination and cultivation [11,12] as well as systematic and phylogenetic advancements [13,14,15] within the species. Native peoples used the plant and/or plant decoctions to treat various ailments and symptoms, including colds, pneumonia, toothaches, stomachaches, fevers, and to address cuts, sores, swellings, and bruises [16,17,18,19]. Bossowey, the Paiute and Shoshone name for *O. occidentalis*, root was used in a tea for treating heavy respiratory issues [17] or was ground up and smoked to treat colds [18]. The roots and seeds were used for food flavoring and the stems were used as a dye for garments [19]. Ethnobotanical literature cites that while both the root and above-ground portions of the plant were used by native peoples, the root was used more often than any other plant part [16,17,18,19].

Little modern research has been done to investigate or substantiate the purported benefits of using *O. occidentalis* plant material or decoctions, apart from solvent extracts that have isolated falcarindiol and a related polyyne [20]. Falcarindiol is present in other plants within the Apiaceae family and has known antifungal properties. The small amount of modern research on active ingredients from *O. occidentalis* is in stark contrast to the well-documented composition and investigation of active ingredients from other aromatic plants within the Apiaceae family, including ajowan, angelica, anise, caraway, carrot, celery, coriander, cumin, dill, and fennel [21,22,23,24,25,26,27,28,29,30].

The current study establishes for the first time, to the best knowledge of the authors, the essential oil composition and stable isotope profile of prominent volatile compounds extracted from *O. occidentalis*. Staying in accordance with guidelines from the research permit issued for this study, only the above-ground portions of the *O. occidentalis* plant were used. Distillation yields are also reported. Results provide insight into the chemical composition and provide fundamental data for substantiation of ethnobotanical applications.

## 2. Results

The essential oil yield (*w/w*) was on average 0.12%. Distillation details and yield data are given in Table 1.

Following steam distillation, 33 volatile compounds were detected by GC/MS and GC/FID (Table 2). Quantified and identified compounds comprise, on average, 99.0% of the essential oil profile. Prominent volatile compounds include methyl chavicol (avg. 61.6%), (*Z*)-β-ocimene (avg. 14.7%), sabinene (avg. 10.5%), and γ-terpinene (avg. 2.8%), comprising approximately 90% of the essential oil profile.

The stable isotope profile of the four most prominent compounds (sabinene, (*Z*)-β-ocimene, γ-terpinene, and methyl chavicol) is detailed in Table 3.

## 3. Discussion

The essential oil yield of the above-ground portions of *Osmorhiza occidentalis* varies from other aromatic plants in the Apiaceae family. Typically, the essential oils extracted from the seed/fruit of plants in the Apiaceae family tend to have higher yields than that of the vegetative plant parts. Within the Apiaceae family, the low end of seed/fruit yield is 0.5 and 1.7% (carrot seed, fennel seed), and on the high end, is 2.8% and 5.3% (anise seed, cumin seed) [23,25,28,30]. Distillation of the vegetative plant parts of other plants in the Apiaceae family is comparable to that of *O. occidentalis* (avg. 0.12%). The low end of vegetative parts yield in the Apiaceae family is 0.2% and 0.3% (celery stems, dill aerial parts), and on the high end, is 0.6% (celery leaves) [26,29]. The modest yield may account for *O. occidentalis* not being previously cultivated for essential oil production. Staying in accordance with guidelines from the research permit issued for this study, only above-ground portions of the *O. occidentalis* plant were used. The essential oil yield from the root may differ from that of the above-ground portions of the plant. This is an area that merits future investigation.

Gas chromatography (GC/MS, GC/FID) analysis resulted in the detection of 33 volatile compounds, although 4 compounds (sabinene, (*Z*)-β-ocimene, γ-terpinene, methyl chavicol) comprise approximately 90% of the essential oil profile. Prominent volatile compounds found in the above-ground portions of *O. occidentalis* are also commonly found in other aromatic plants in the Apiaceae family. Reports of these prominent volatile compounds and the plants they are found in include sabinene (3.3% in carrot seed) [25], γ-terpinene (23.5 to 24.8% in ajowan; 3.8 to 5.4% in cumin seed) [21,28], methyl chavicol (0.6 to 1.4% in anise seed; 50.2 to 69.2% in fennel seed) [23,30], and (*E*)-anethole (81.5 to 91.3% in anise seed) [23]. While the essential oil profile of the above-ground portions of *O. occidentalis* is not entirely unique, the abundance of (*Z*)-β-ocimene (avg. 14.7%) is rare in essential oils within the Apiaceae family [21,22,23,24,25,26,27,28,29,30,32].

Compared to GC/FID and GC/MS, GC/IRMS is a relatively novel application for essential oil analysis. However, previous stable isotope research has been conducted on several essential oils in the Apiaceae family, including anise, coriander, dill, and fennel [33,34,35], albeit for different volatile compounds than those analyzed in the current study. Since GC/IRMS is not typically performed on all compounds comprising an essential oil, it is logical to compare stable isotope values performed on the same volatile compounds previously researched, despite the plant family they originated from. When comparing stable isotope values from the current study to the same compounds found in C_3_ carbon fixing plants from the Lamiaceae and Asteraceae families, some values differ, while others fall within previously established ranges of natural products. *δ*^2^H values for (*Z*)-β-ocimene and γ-terpinene differ when comparing the current study (−356.068 to −342.271; −391.879 to −383.912) to previously established values for the same compounds (−299.953 to −272.945 in Peruvian basil; −285 to −217 in thyme and oregano), respectively [36,37]. *δ*^2^H values for methyl chavicol are within previously established ranges when comparing the current study (−175.225 to −169.497) to previously established values for the same compound (−193 to −105 in tarragon and basil) [38]. *δ*^13^C for (*Z*)-β-ocimene and methyl chavicol are also within previously established ranges when comparing the current study (−31.261 to −30.291; −36.360 to −35.695) to previously established values for the same compounds (−34.969 to −30.309 in Peruvian basil; −36.4 to −28.8 in tarragon and other naturals), respectively [36,38].

Both the ethnobotanical applications and essential oil profile of the above-ground portions of *O. occidentalis* suggest that this plant may be another species within the Apiaceae family with future medicinal and commercial value. However, additional studies need to be done regarding potential antibacterial, antifungal, antioxidant, and other medically related properties. Additionally, while the essential oil yield determined in the current study is modest, other cultivation methods and extraction techniques may improve the yield. Future research should focus on these aspects, as well as the essential oil extraction from the plant roots, and not exclusively the above-ground portions of the plant.

## 4. Materials and Methods

*Osmorhiza occidentalis* plant material was collected on July 8, 2022, from native populations located on public lands (Bureau of Land Management) in Tooele County, Utah, USA (40°28′3″ N 112°10′32″ W; 2798 m elevation). In accordance with guidelines from the research permit issued for this study, only the above-ground portions of the plant were used. Above-ground portions were cut from flowering plants and divided into three groups to determine weight, yield, the composition of the extracted essential oil, and stable isotope profile of prominent volatile compounds. For simplicity and consistency, each sample is referred to by a letter, A–C. Representative voucher samples are held in the Utah Valley University Herbarium (UVSC) and Young Living Aromatic Herbarium (YLAH): *O. occidentalis* Torr., Wilson 2022-01 (UVSC) and *O. occidentalis* Torr., Wilson 2022-02 (YLAH), respectively.

Plant material was prepared for laboratory-scale distillation as follows: the above-ground portions were separated into three groups, bagged, and stored at −20 ± 2 °C until steam distilled. Steam distillation was performed in triplicate, resulting in 3 distillations over the course of this project.

Laboratory-scale distillation was as follows: 1.5 L of water was added to 2 L steam generator that fed into a 2 L distillation chamber, plant material was accurately weighed and added to the distillation chamber, distillation for 1.5 h from pass-over by indirect steam, essential oil separated by a cooled condenser and Florentine flask. Essential oil samples were each filtered and stored at room temperature in a sealed amber glass bottle until analysis.

The percent yield was calculated as the ratio of the mass of processed plant material immediately before distillation to the mass of essential oil produced, multiplied by 100.

Essential oil samples were analyzed, and volatile compounds identified, by GC/MS using an Agilent 7890B GC/5977B MSD (Agilent Technologies, Santa Clara, CA, USA) and Agilent J&W DB-5, 0.25 mm × 60 m, 0.25 μm film thickness, fused silica capillary column. Operating conditions: 0.1 μL of sample (20% soln. for essential oils in ethanol), 100:1 split ratio, initial oven temp. of 40 °C with an initial hold time of 5 min., oven ramp rate of 4.5 °C per min. to 310 °C with a hold time of 5 min. The electron ionization energy was 70 eV, scan range 35–650 amu, scan rate 2.4 scans per sec., source temp. 230 °C, and quadrupole temp. 150 °C. Volatile compounds were identified using the Adams volatile oil library [31] using Chemstation library search in conjunction with retention indices. Note that limonene/β-phellandrene elute as a single peak. Their amounts were determined by the ratio of masses 68 and 79 (limonene), 77 and 93 (β-phellandrene). Volatile compounds were quantified and are reported as a relative area percent by GC/FID using an Agilent 7890B GC and Agilent J&W DB-5, 0.25 mm × 60 m, 0.25 μm film thickness, fused silica capillary column. Operating conditions: 0.1 μL of sample (20% soln. for essential oils in ethanol, 1% for reference compounds in ethanol, 0.1% soln. for C7–C30 alkanes in hexane), 25:1 split ratio, initial oven temp. of 40 °C with an initial hold time of 2 min., oven ramp rate of 3.0 °C per min. to 250 °C with a hold time of 3 min. Essential oil samples were analyzed in triplicate by GC/FID to ensure repeatability (standard deviation ≤ 1 for all compounds). Compounds were identified using retention indices coupled with retention time data of reference compounds (MilliporeSigma, Sigma-Aldrich, St. Louis, MS, USA).

The hydrogen and carbon stable isotope ratios of essential oils were analyzed by GC/IRMS using a Thermo TRACE 1310 GC coupled to a Thermo Delta V Advantage Isotope Ratio Mass Spectrometer (ThermoFisher Scientific, Waltham, MA, USA), with an Agilent J&W DB-5, 0.25 mm × 60 m, 0.25 μm film thickness, fused silica capillary column.

Essential oil samples were prepared for GC/IRMS analysis as follows: 35 mg of sample was weighed into a 2 mL clear glass vial and brought up to 1 mL with hexane. A 100 μL aliquot was removed and placed into a second vial, which was then brought up to 1 mL with hexane and used for ^2^H/^1^H analysis. From the second sample vial, a 90 μL aliquot was removed and placed into a third vial, brought to 1 mL in hexane, and used for ^13^C/^12^C analysis.

GC operating conditions are as follows: splitless injection of 1 μL of sample with splitless time set at 0.25 min., injection port 270 °C, initial oven temp. 50 °C with an initial hold time of 2.0 min., oven ramp rate of 6.0 °C per min. to 250 °C with a hold time of 2.0 min., then an oven ramp rate of 10.0 °C per minute to 310 °C with a hold time of 7.0 min., helium carrier gas with constant flow 1.55 mL/min. After passing through the capillary column, samples were sent through the HTC reactor for ^2^H/^1^H analysis or the combustion reactor for ^13^C/^12^C analysis. HTC reactor temp. was set to 1420 °C and was regularly conditioned by injecting 1 μL of hexane in backflush mode. Combustion reactor temp. was set to 1000 °C and was conditioned with oxygen at regular intervals.

To normalize IRMS results, reference materials were purchased from Dr. Arndt Schimmelmann at Indiana University and from the United States Geological Survey (USGS) – Reston Stable Isotope Laboratory. *δ*^2^H isotope ratios are expressed relative to VSMOW and *δ*^13^C isotope ratios to VPDB. The following three reference materials, along with their known values, were used to normalize results: hexadecane #C (USGS69), *δ*^2^H: 381.4‰, *δ*^13^C: −0.57‰; nonadecane #2, *δ*^2^H: −56.3‰, *δ*^13^C: −31.99‰; and tetradecanoic acid methyl ester #14M, −231.2‰, *δ*^13^C: −29.98‰. 

Samples were analyzed in triplicate to ensure repeatability. Isotope ratios were determined for the following prominent compounds: sabinene, *cis*-β-ocimene, γ-terpinene, and methyl chavicol. *δ*^2^H values are reported with a standard deviation ≤ 2.6‰ and *δ*^13^C values are reported with a standard deviation ≤ 0.2‰.

## Figures and Tables

**Table 1:** Yield data, including mass of plant material distilled (g), essential oil yield (g), and calculated yield (%) from *Osmorhiza occidentalis* samples (*n* = 3).

Sample	Plant Mass Distilled (g)	Yield EO (g)	Yield EO (%)
A	148.95	0.15	0.10
B	147.39	0.18	0.12
C	146.55	0.18	0.12
Avg:	147.63	0.17	0.12
%RSD (*n* = 3)			10.91

**Table 2:** Aromatic profile of *Osmorhiza occidentalis* essential oil samples A–C. Reported values below represent averages from samples analyzed in triplicate, which was done to ensure repeatability (standard deviation ≤ 1 for all compounds). Values less than 0.1% are denoted as trace (t) and those not detected in that sample as nd. Unidentified compounds less than 0.1% are not included. KI is the Kovat’s Index value and was previously calculated by Robert Adams using a linear calculation on a DB-5 column [31]. Relative area percent was determined by GC/FID.

KI	Compound	A	B	C
846	(2*E*)-hexenal	t	0.1	0.1
924	α-thujene	0.1	0.1	0.1
932	α-pinene	0.3	0.1	0.2
946	camphene	t	t	t
969	sabinene	9.1	13.6	8.8
974	β-pinene	0.1	0.1	0.1
988	myrcene	0.7	0.7	0.7
1002	α-phellandrene	0.5	t	0.1
1008	δ-3-carene	t	t	t
1014	α-terpinene	0.1	t	t
1020	o-cymene	2.4	2.8	2.8
1024	limonene	0.1	0.1	0.1
1025	β-phellandrene	0.6	t	0.1
1032	(*Z*)-β-ocimene	18.0	11.6	14.4
1044	(*E*)-β-ocimene	0.7	0.5	0.6
1054	γ-terpinene	3.6	2.2	2.5
1065	*cis*-sabinene hydrate	t	t	t
1086	terpinolene	0.5	0.3	0.2
1095	linalool	0.2	0.4	0.4
1100	n-nonanal	t	t	t
1128	allo-ocimene	2.9	1.8	2.3
1141	camphor	t	t	nd
1148	citronellal	t	t	t
1174	terpinen-4-ol	0.1	0.1	0.1
1186	α-terpineol	t	t	t
1195	methyl chavicol	57.9	63.0	63.9
1223	citronellol	0.1	0.1	0.1
1232	thymol methyl ether	t	t	nd
1235	neral	nd	nd	t
1282	(*E*)-anethole	0.9	0.9	1.1
1392	(*Z*)-jasmone	nd	t	nd
1403	methyl eugenol	0.2	0.2	0.1
1430	β-copaene	0.1	t	0.1
Total		99.1	98.9	98.9

**Table 3:** Stable isotope ratios, *δ*^2^H and *δ*^13^C, for sabinene, *cis*-β-ocimene, γ-terpinene, and methyl chavicol. Samples were analyzed in triplicate to ensure repeatability (*δ*^2^H values are reported with a standard deviation ≤2.6‰ and *δ*^13^C values are reported with a standard deviation ≤0.2‰). *δ*^2^H isotope ratios are expressed relative to VSMOW and *δ*^13^C isotope ratios to VPDB.

	Sabinene		(*Z*)-β-Ocimene		γ-Terpinene		Methyl Chavicol	
	*δ*^2^H (‰)	*δ*^13^C (‰)	*δ*^2^H (‰)	*δ*^13^C (‰)	*δ*^2^H (‰)	*δ*^13^C (‰)	*δ*^2^H (‰)	*δ*^13^C (‰)
A	−402.257	−32.734	−342.271	−31.261	−383.912	−35.429	−169.827	−36.360
B	−378.893	−33.461	−356.068	−30.908	−391.879	−34.342	−169.497	−35.695
C	−399.287	−33.462	−354.323	−30.291	−390.927	−33.443	−175.225	−35.816

## Data Availability

The data presented in this study are available upon request from the corresponding author.
